# Advancing Structure-Based Drug Development: A Rebuild of the IMCA-CAT Beamline at the Advanced Photon Source

**DOI:** 10.1063/4.0001166

**Published:** 2025-10-27

**Authors:** Melissa Carrillo, John P. Bacik, Erica Duguid, J. Lewis Muir, Clark Williams, Andrew Mayton, Kevin D'Amico, Stephan Ginell, Nathan Brown, Lisa J. Keefe

**Affiliations:** 1 Industrial Macromolecular Crystallography Association Collaborative Access Team (IMC-CAT) Sector 17-ID, Lemont, IL, USA; 2 137partners, LLC, Bridgton, ME, USA; 3 Consultant, Naperville, IL, USA; 4 AIM Concepts, Littleton, CO, USA

## Abstract

IMCA-CAT is a structural biology beamline located at the Advanced Photon Source (APS) that caters to pharmaceutical companies for drug discovery. During the 2023-2024 APS-U shutdown, IMCA-CAT undertook a comprehensive beamline upgrade - essentially rebuilding the beamline to enhance performance and automation. The upgrade focused on integrating advanced optics, including a set of two focusing mirrors configured in a Kirpatrick-Baez configuration, compound refractive lenses (CRLs), and a monochromator from Axilon AG. These state-of-the-art components provide improved microfocusing capabilities and enable rapid beam size changes with minimal flux loss, optimizing conditions for a wide range of experimental needs. To address the growing demand for high-throughput data collection, IMCA-CAT collaborated with Rigaku to upgrade its robotic sample handler and implement larger dewars capable of holding 672 samples. A joint development effort also led to the creation of a basket-based puck loading system, significantly reducing downtime during set-ups. In addition, IMCA-CAT partnered with AIM Concepts to develop a new XYZ-axis goniometer, offering improved precision in sample rotation and translation. The combined upgrades of faster robotics, enhanced sample handling, and precise positioning streamline data collection and reduce experimental time, allowing for a doubling of throughput to 1,000 datasets per day. Together, these advancements position IMCA-CAT at the forefront of automation-driven structural biology. The upgraded beamline is well equipped to accelerate drug discovery and development, reinforcing IMCA-CAT’s pivotal role in the future of protein crystallography.

